# Thiamine Deficiency Secondary to Pain-Induced Oral Aversion

**DOI:** 10.1177/00099228241286239

**Published:** 2024-10-13

**Authors:** Bryan Cusack, Dustin Paul, Puneet Kochar, Talbot Weston, Jacob Nelsen, Keith E. Williams

**Affiliations:** 1 Milton S. Hershey Medical Center, Penn State Health, Hershey, PA, USA

## Introduction

As there is little endogenous synthesis of thiamine, humans depend entirely on dietary intake of this vitamin. Humans also have limited ability to store thiamine and persons not ingesting adequate thiamine can show signs of deficiency in as few as 18 days.^1^ Thiamine serves as a cofactor for several enzymatic reactions involved in energy metabolism essential to neuronal development and cerebral metabolism.^2^ In addition, thiamine-dependent enzymes regulate biosynthesis of neurotransmitters such as acetylcholine, gamma-aminobutyric acid (GABA), and reducing substrates used in neuronal oxidant stress defenses. Thiamine deficiency (TD) decreases the activity of thiamine-dependent enzymes and triggers a sequence of metabolic events leading to energy compromise and neuronal cell death.^3^ Progressive neurodegeneration can lead to a sequela known as Wernicke’s encephalopathy (WE). Classically, the presentation of WE is characterized by a triad of ataxia, altered mental status, and nystagmus or ophthalmoplegia; however, in pediatric populations, presentation varies based on localization of lesions.^4^

In school-aged children, TD has been most often identified as being the result of a critical illness resulting in more rapid depletion of thiamine due to increased metabolic demands secondary to the illness as well as an illness-related decline in oral intake.^5^ Congenital anomalies affecting thiamine metabolism is also a cause of TD in children. Defects in 4 genes, SLC19A2, SLC19A3, SLC25A19, and TPK1, lead to TDs. Two of these genes, SLC19A2 and SLC19A3, are involved in absorbing thiamine in the lower intestine, while 1 gene, SLC25A19, is involved in the absorption of thiamine into the mitochondria. The TPK1 serves to activate thiamine within the cell. A defect in SLC19A2 may include hearing loss and megaloblastic anemia.^6^ Thiamine supplementation has resulted in better control of anemia in many patients with this defect. Defects in SLC19A3, SLC25A19, and TPK1 may result in recurrent encephalopathy, basal ganglia necrosis, generalized dystonia, severe disability, and early death. Although there is great variability in the severity of these congenital deficiencies, thiamine supplementation has been shown to improve clinical outcome and survival.^6^ TD has also been reported in adolescents with eating disorders, usually anorexia nervosa, often because of limited intake.^7^ Less often, children with extremely limited diets have been diagnosed with TD. This has been found among children with autism spectrum^8^ as well as neurotypical children.^9^ We review the clinical course of a 12-year-old male with self-limited dietary intake who developed WE, discuss factors related to the development of the deficiency, and provide suggestions to prevent similar cases.

## Case Presentation

A 12-year-old male, whose previous history included only mild asthma that did not require medication, arrived as an outside hospital transfer where he was noted to have severe malnutrition, oculomotor dysfunction, generalized weakness, and gait instability. Six weeks prior to admission, the patient underwent a palate expander fixation. Prior to this procedure, his body mass index (BMI) was at the 97th percentile, and he had no history of psychiatric or developmental disorders. After this procedure, he stopped talking and developed an aversion to eating all solids secondary to oral pain. Although the expander was removed after 26 days, the food refusal continued and resulted in a 19.5 kg weight loss (28% of his body weight). After his initial refusal to eat solids, his pediatrician recommended oral supplementation with PediaSure (Abbott Nutritionals, Abbott Park, Illinois). Although he was scheduled for weekly weight checks at his pediatrician’s office, multiple visits were missed due to transportation issues. As a result of his worsening left eye ptosis, generalized fatigue, and persistent weight loss, he presented to the emergency department of an outside hospital. Outside laboratory evidence was remarkable for a mild hypokalemia (2.9) and hypophosphatemia (3.1). The physical exam was significant for oral candidiasis, horizontal nystagmus with lateral gaze, photophobia, left ptosis, and right upper quadrant abdominal tenderness with voluntary guarding. Abdominal exam findings, supported by abdominal ultrasound, revealed findings suggestive of hepatic steatosis. However, all other outside imaging, including a computed tomography (CT) brain, magnetic resonance imaging (MRI) brain, and magnetic resonance angiography (MRA) head/neck with and without contrast, did not reveal any acute intracranial abnormalities. The patient was transferred to our hospital for further evaluation and management.

On admission, the patient was afebrile, heart rate 95 bpm, blood pressure 123/85 mm Hg, respirations 20 breaths per minute, and saturating 100% on room air. Neurological physical examination findings were notable for the following: left eye ptosis and on leftward gaze was only able to abduct 50% and rightward gaze was able to abduct 60%, limited upward gaze and fatigable left eye after 30 seconds, and no diplopia. He was also found to have white-yellow oral plaques on the tongue and posterior oropharynx, which were easily scraped off the tongue; however, the pain made him unable to tolerate his own secretions. Otherwise, he had a normal cardiopulmonary exam, 5/5 musculoskeletal strength throughout, and 2+ reflexes.

**Figure 1.**
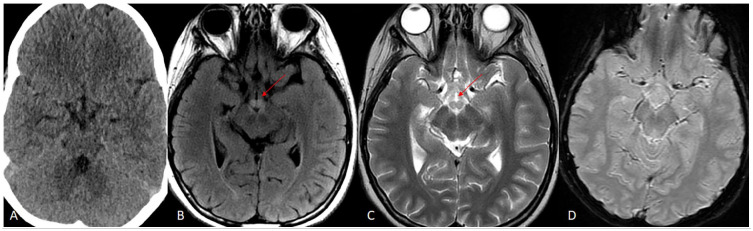
Initial CT scan without contrast is negative. Subsequently performed MRI brain on the same day demonstrates T2/FLAIR hyperintense signal in bilateral mammillary bodies (images A and B; red arrows). No associated gradient blooming to represent hemorrhage.

**Figure 2.**
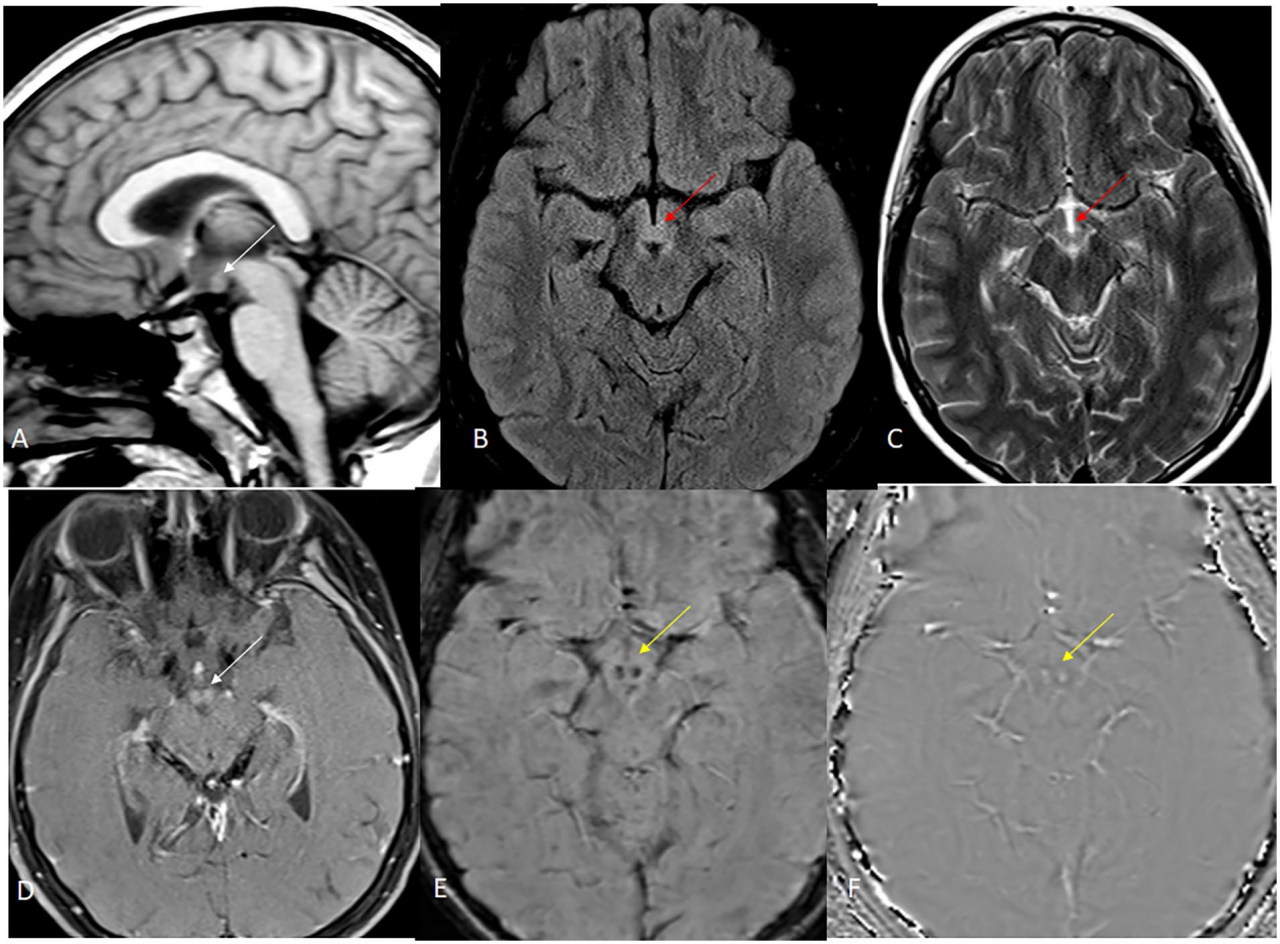
Repeat MRI performed 2 days later in our hospital demonstrates mild swelling of the mammillary bodies without T1 hyperintensity (image A, white arrow) with abnormal T2/FLAIR hyperintensity (images B and C; red arrows). Post-contrast image demonstrates mild enhancement (image D, white arrow). SWI/phase images show hemorrhage bilaterally (images E and F; yellow arrows).

At the time of admission, his diet consisted of two to three 8-ounce containers of PediaSure he consumed via straw as well as small amounts of water and apple juice. Given his degree of odynophagia and oral aversion, a nasogastric (NG) tube was placed, and Nutrition Services were consulted. He was started on Nutren Junior with Fiber (Vevey, Switzerland), which was titrated to a caloric goal of 2000 kcal/day. For his oropharyngeal candidiasis, he was started on fluconazole via NG tube with the addition of oral Nystatin. Daily comprehensive metabolic panels were obtained to monitor for electrolyte derangements given his risk of refeeding syndrome, and derangements were repleted, as necessary. Pediatric neurology was consulted to further evaluate his nystagmus, ptosis, and reported gait instability. Initial differential diagnosis consisted of Myasthenia Gravis, Miller Fisher Syndrome, Multiple Sclerosis, Lyme Disease, and vitamin deficiencies. After a thorough examination, they recommended a test for acetylcholine binding, blocking, and modulating antibodies, as well as an ophthalmology consult to evaluate autoimmune and ocular pathologies. Ophthalmology evaluation confirmed bilateral abduction and adduction deficits with roaming nystagmus concerning for a central process rather than a primary ocular cause. The MRI studies of the brain and orbits with and without contrast were obtained and demonstrated subtle signal intensity and enhancement involving the mammillary bodies and hypothalamus (Figures 1 and 2). Given the imaging results and the patient’s clinical features, a diagnosis of WE was established. He was started on a 5-day course of intravenous (IV) thiamine 200 mg 3 times daily. High-dose IV thiamine administration has been shown to be efficacious in swift repletion of thiamine stores and optimal improvement in WE.^10^ Although oral administration was not feasible with this child due to limited intake, oral thiamine would not have been optimal due to lower bioavailability. Parenteral administration of thiamine bypasses the decreased gastrointestinal absorption of acutely ill patients and maximizes bioavailability. After he had received 2 days of IV thiamine as well as thiamine from the oral supplement, his thiamine level was 108 (B1; normal 70-180). Other vitamin levels were also obtained, including pyridoxine 20.3 (B6; normal 20-125), folate 11.9 (B9; normal >5.9), cobalamin 211 (B12; normal 211-946), 25-hydroxy vitamin D level 8 (deficiency <20). His vitamin D deficiency was addressed both with Nutren Jr and a liquid vitamin delivered via NG tube. By the end of a 6-day hospitalization, the left-sided ptosis, bilateral nystagmus, gait ataxia, and generalized weakness had resolved. Although he was talking and more interactive, he continued to have problems with swallowing and received most of his nutrition via NG tube. Discharge medication included 100 mg oral thiamine (via NG tube) and fluconazole. At 6-week follow-up with gastroenterology, he was eating 3 meals and 2 snacks daily and the NG tube had been removed. Parents reported he started eating with resolution of oral candidiasis. At 12-week follow-up with Pediatric Neurology, there was no ptosis, nystagmus, or gait issues. Speech was normal, and parents reported he had returned to baseline functioning. Written permission for this case study was obtained by the child’s parents. This case study was exempt from Institutional Review Board (IRB) review.

## Discussion

Although the neurodegeneration of WE is subtle, an inciting trigger with hypermetabolic state, such as acute infection or refeeding following malnutrition, commonly occurs prior to onset of symptoms.^11^ In this patient, we expect a cascade of events led to the development of the thiamine deficiency and subsequent WE. Pain secondary to insertion of a palate expander resulted in food refusal, which continued even after the removal of the expander because the pain resulted in an oral aversion. The on-going aversion resulted in dramatic weight loss. The resulting malnutrition caused oral, and perhaps pharyngeal and esophageal candidiasis, which maintained the food refusal and interfered with nutritional interventions.

We believe there are several lessons to be learned from this case which might be applied in similar situations. In this case, the patient’s deterioration was missed due to the caregiver’s reliance on public transportation to follow-up appointments. Classic management of pediatric weight loss includes weight checks at regular and frequent intervals to assess response to dietary supplementation. This model of weight management may not be possible with patients whose transportation is uncertain. Following the COVID-19 pandemic, the use of telehealth services has become commonplace in clinical patient care. Considering virtual weight checks using weights collected in the patient’s home for this population would allow for improvements in continuity of care and rapid reassessment of malnutrition management. To address this child’s food refusal and weight loss, complete oral supplements, in this case, PediaSure, were recommended. Despite daily ingestion of 500 to 750 mL of this oral supplement, his thiamine was depleted to the point of developing WE. The daily recommended intake of thiamine for a child 9 to 13 years is 0.9 mg. One 250 mL serving of PediaSure provides 0.30 mg of thiamine.^12^ Although 3 containers of PediaSure would have provided the recommended amount of thiamine for his age, he did not always drink 3 containers daily and he had already experienced some weight loss and thiamine depletion was already underway. It has been suggested malnourished children require thiamine doses between 10 and 30 mg daily to prevent clinical deterioration; thus, this child probably required thiamine supplementation beyond what was available in this supplement even with increased consumption.^7^ Although the patient did not initially present with neurologic symptoms concerning suggestive of WE, a review of available commercial 100 mg thiamine supplements—dosing that is proven to be capable of reducing complications of malnutrition and thiamine deficiency—shows that a 120-day supply costs an average of $10.^2^ Even with a low index of suspicion, consideration for preventable complications of malnutrition should be assessed to reduce overall health care expenditures for the patient.

## Author Contributions

**DP:** Contributed to conception, design, and interpretation; Contributed to drafting and revising manuscript.

**BC:** Contributed to conception, design, and interpretation; Contributed to drafting and revising manuscript.

**PK:** Contributed to acquisition and interpretation; Contributed to drafting and revising manuscript.

**TW:** Contributed to acquisition and interaction; Contributed to drafting and revising manuscript.

**JN:** Contributed to acquisition, drafting and revising manuscript.

**KEW:** Contributed to design and analysis; Contributed to drafting and revising manuscript.
